# A ROS‐Responsive Dual‐Targeting Drug Nanocarrier Serving as a GSI Synergist and Ferroptosis Sensitizer for T‐Cell Acute Lymphoblastic Leukemia

**DOI:** 10.1002/advs.202505087

**Published:** 2025-05-31

**Authors:** Ruinan Jia, Yang Liu, Jilong Xiao, Yuan Xia, Xin Zhao, Huixian Ma, Jingjing Ye, Zhiyue Zhang, Tao Sun, Chunyan Ji

**Affiliations:** ^1^ Department of Hematology Qilu Hospital Cheeloo College of Medicine Shandong University Jinan 250012 P. R. China; ^2^ Shandong Key Laboratory of Hematological Diseases and Immune Microenvironment Qilu Hospital Shandong University Jinan 250012 P. R. China; ^3^ NMPA Key Laboratory for Technology Research and Evaluation of Drug Products Key Laboratory of Chemical Biology (Ministry of Education) Shandong Key Laboratory of Targeted Drug Delivery and Advanced Pharmaceutics Department of Pharmaceutics School of Pharmaceutical Sciences Cheeloo College of Medicine Shandong University Jinan Shandong 250012 P. R. China; ^4^ Cryomedicine Laboratory Qilu Hospital of Shandong University Jinan Shandong Province 250012 China

**Keywords:** γ‐secretase inhibitors (GSIs), dihydroartemisinin (DHA), ferroptosis, T‐cell acute lymphoblastic leukemia (T‐ALL)

## Abstract

T‐cell acute lymphoblastic leukemia (T‐ALL) is a highly aggressive hematological malignancy for which targeted therapies remain underdeveloped. Oncogenic mutations in Notch1 occur in up to 75% of T‐ALL patients. Although γ‐secretase inhibitors (GSIs) can block Notch1 activation, their clinical application is limited by side effects and reduced sensitivity. Here, a self‐assembling, reactive oxygen species (ROS)‐responsive nanotherapeutic strategy—PHD/G‐NPs—co‐loaded with GSI and controlled released dihydroartemisinin (DHA), and modified with a CD38 antibody is reported. The CD38 antibody specifically targets T‐ALL cells, while GSI selectively inhibits Notch1, resulting in a dual‐targeting approach. GSI is released first, inhibiting Notch1 activation and inducing the death of a subset of T‐ALL cells. To eliminate semi‐quiescent T‐ALL cells that escape initial therapy by elevating ROS levels, a ROS‐sensitive DHA delivery system is employed to enhance ferroptosis and boost GSI efficacy. After elucidating the mechanism of action of PHD/G‐NPs in T‐ALL cells, PHD/G‐NPs are combined with αPD‐1, which triggers an anti‐tumor immune response in vivo. This dual‐targeting strategy using CD38‐modified PHD/G‐NPs enables controlled drug release, enhances ferroptosis, mitigates GSI‐induced gastrointestinal toxicity, and improves therapeutic efficacy. This nanomedical approach offers a novel strategy for targeted T‐ALL treatment.

## Graphical abstract

A self‐assembling nanotherapeutic strategy is designed—CD38‐PHD/G‐NPs, which released γ‐secretase inhibitor first, followed by adaptive dihydroartemisnin release in response to elevated reactive oxygen species levels. This controlled, sequential release strategy maximizes the synergistic anti‐leukemic efficacy of two agents with distinct mechanisms of action—simultaneously targeting Notch1‐mutated T‐cell acute lymphoblastic leukemia cells and compensating for each other's limitations.

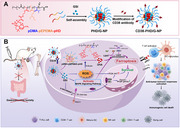

## 1 Introduction

T‐cell acute lymphoblastic leukemia (T‐ALL) is a highly aggressive hematological malignancy that arises from the malignant transformation of T‐cell progenitors.^[^ ^1^ ^]^ Compared with B precursor ALL and myeloid leukemia, T‐ALL is associated with higher relapse rates and poorer prognoses.^[^ ^2^, ^3^ ^]^ The 5‐year survival rate for T‐ALL patients under 60 years old is only 40–50%, with an even worse outlook for older patients.^[^ ^4^ ^]^ Approximately 50% of adult T‐ALL patients exhibit resistance to first‐line treatments, and the development of personalized targeted therapies remains limited.^[^ ^5^, ^6^ ^]^

The most common genetic alteration in T‐ALL is an oncogenic mutation in Notch1, found in up to 75% of patients. This mutation leads to ligand‐independent activation of Notch1, promoting uncontrolled proliferation and cell survival.^[^ ^7^, ^8^, ^9^ ^]^ Notch1 plays a critical role in hematopoietic cell fate determination, which explains the strong selection pressure for oncogenic Notch mutations in T‐ALL.^[^ ^10^ ^]^ Due to its high mutation frequency, Notch1 is an attractive therapeutic target, spurring the development of small‐molecule inhibitors aimed at the Notch1 signaling pathway. The most widely used of these is the γ‐secretase inhibitor (GSI), which blocks the proteolytic cleavage of Notch, thereby preventing downstream activation.^[^ ^11^ ^]^ However, both preclinical and clinical studies have shown that GSIs lack sustained anti‐leukemic activity, typically causing only transient growth arrest rather than cell death.^[^ ^12^ ^]^ Giulia et al. identified that, under GSI treatment, reactive oxygen species (ROS) scavenging enzymes are upregulated in a subset of cells, leading to elevated intracellular ROS levels. These cells adopt a semi‐quiescent state that allows survival under high ROS conditions and GSI‐induced stress. This alternative state is characterized by low replication stress, enhanced genome stability, and resistance to cell death.^[^ ^13^ ^]^ Thus, while GSI inhibits proliferation, semi‐quiescent T‐ALL cells can persist by adapting to oxidative stress and activating survival pathways. Moreover, GSI is associated with significant toxicity in normal tissues, most notably causing severe gastrointestinal side effects such as diarrhea.^[^ ^14^, ^15^ ^]^

Dihydroartemisinin (DHA) is a semi‐synthetic derivative of the natural compound artemisinin and has been reported to inhibit the growth of AML, glioblastoma, and liver cancer.^[^ ^16^, ^17^, ^18^, ^19^ ^]^ DHA contains an endoperoxide bridge, which can be cleaved by Fe(II), leading to the formation of toxic free radicals.^[^ ^16^ ^]^ DHA induces cell death primarily through ferroptosis, a form of regulated cell death triggered by lipid peroxidation.^[^ ^17^, ^20^, ^21^ ^]^ Notably, the anti‐tumor effect of DHA is partially due to its high sensitivity to ROS, which allows it to exert selective cytotoxicity against cancer cells.^[^ ^22^ ^]^ Given the high ROS levels found in semi‐quiescent T‐ALL cells following GSI treatment, and DHA's ROS sensitivity, DHA is a promising candidate to overcome the reduced GSI sensitivity in T‐ALL. However, both GSI and DHA face limitations such as low aqueous solubility, short plasma half‐life, and nonspecific distribution^[^ ^23^, ^24^, ^25^ ^]^ which restrict their therapeutic potential.

**Scheme 1:**
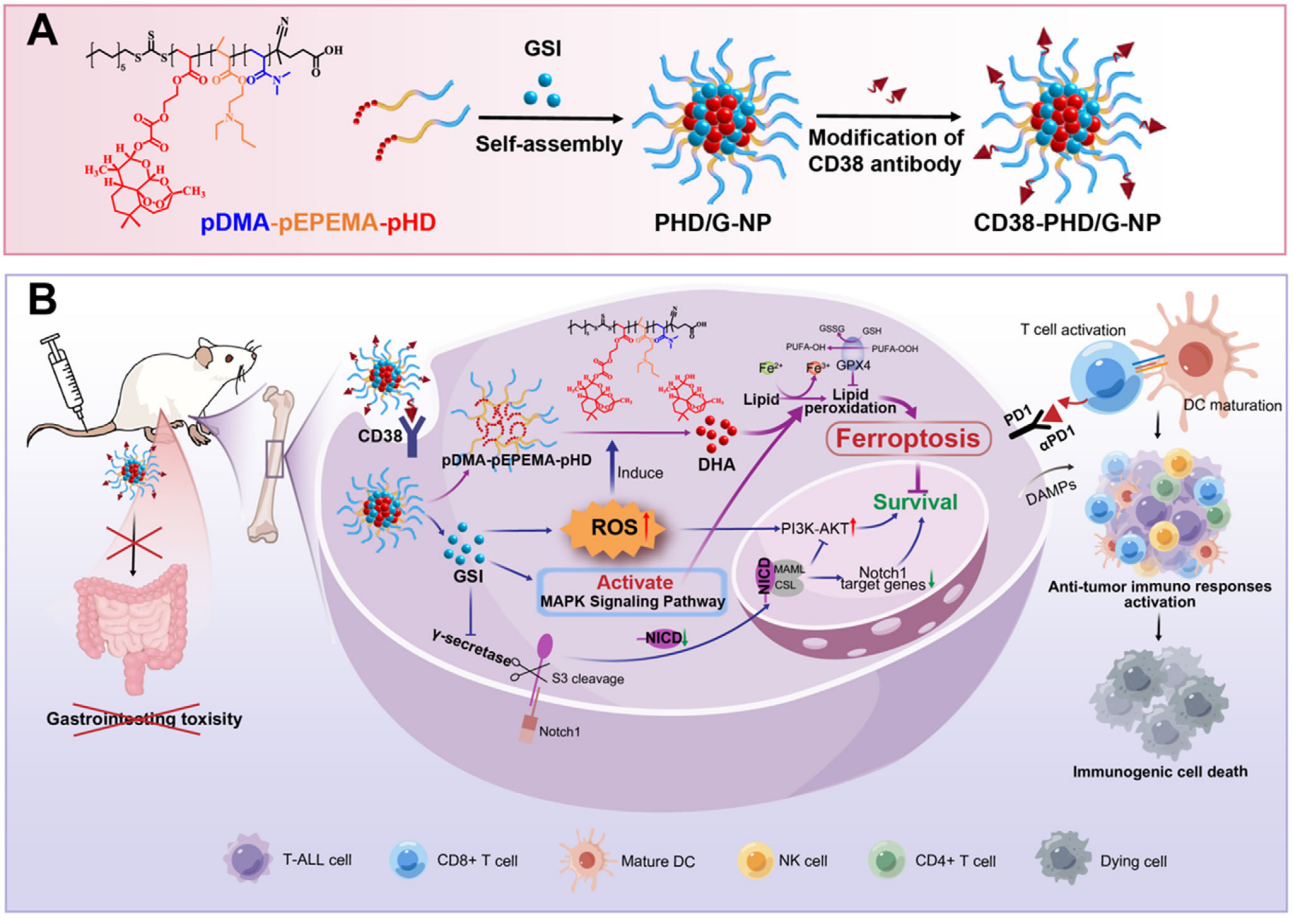
A) The self‐assembling, ROS‐responsive polymer pDMA‐pEPEMA‐pHD (PHD‐NPs) was constructed, featuring a hydrophilic DMA block, a hydrophobic EPEMA block, and a ROS‐responsive bridge linking HEA and DHA. PHD‐NPs were used to encapsulate GSI and subsequently modified with a CD38 antibody to form CD38‐PHD/G‐NPs. B) The CD38 antibody enabled the targeted identification of T‐ALL cells and helped mitigate the severe side effects associated with GSI treatment. GSI inhibited Notch1 activation and induced the death of a subset of T‐ALL cells. In the surviving ROS^high^‐semi‐quiescent T‐ALL cells following GSI treatment, DHA was released via ROS‐responsive bridge degradation. The ferroptosis induced by DHA was further enhanced through activation of the MAPK signaling pathway, which was upregulated post‐GSI treatment. Additionally, damage‐associated molecular patterns (DAMPs) released during ferroptosis, together with αPD‐1 administration, triggered an anti‐tumor immune response.

To address these challenges, we developed a self‐assembling, ROS‐responsive drug nanocarrier (**Scheme** **1**A) designed to treat T‐ALL by acting as both a GSI enhancer and ferroptosis sensitizer. Specifically, we synthesized a ROS‐responsive polymer–drug conjugate, pDMA–pEPEMA–pHD, featuring a hydrophilic DMA block, a hydrophobic EPEMA block, and a ROS‐sensitive linkage between HEA and DHA. This conjugate self‐assembles into nanoparticles (PHD‐NPs) capable of encapsulating GSI to form PHD/G‐NPs. The ROS‐responsive bridge was designed to enhance the activity of DHA and overcome the reduced GSI sensitivity in T‐ALL. Once internalized by T‐ALL cells, PHD/G‐NPs undergo structural changes that facilitate the rapid release of GSI. The released GSI inhibits the production of activated Notch1 (NICD), suppressing cell proliferation. However, a subset of ROS^high^ semi‐quiescent T‐ALL cells persists and activates the MAPK signaling pathway. As ROS levels continue to rise in semi‐quiescent T‐ALL cells, the ROS‐sensitive bridge degrades, releasing DHA in its active form. Notably, the MAPK pathway enhances DHA sensitivity in these resistant cells. DHA then amplifies the cytotoxic effect of GSI by inducing deeper ferroptosis with increasing lipid peroxidation. Moreover, damage‐associated molecular patterns (DAMPs) released during ferroptosis, in combination with αPD‐1 administration, promote dendritic cell (DC) maturation, T‐cell infiltration, and natural killer (NK) cell activation—collectively triggering an in vivo anti‐tumor immune response. This combinatorial strategy acts as an “unarmed counter move,” exhibiting potent efficacy against Notch1‐mutated T‐ALL cells. To reduce the severe side effects associated with GSI, we further modified PHD/G‐NPs with a CD38 antibody. CD38‐PHD/G‐NPs demonstrated effective inhibition of T‐ALL cell growth and improved safety profiles in mouse models (Scheme 1B).

## 2 Results and Discussion

### 2.1 Enhanced GSI Cytotoxicity against T‐ALL Cells by DHA

**Figure 1:**
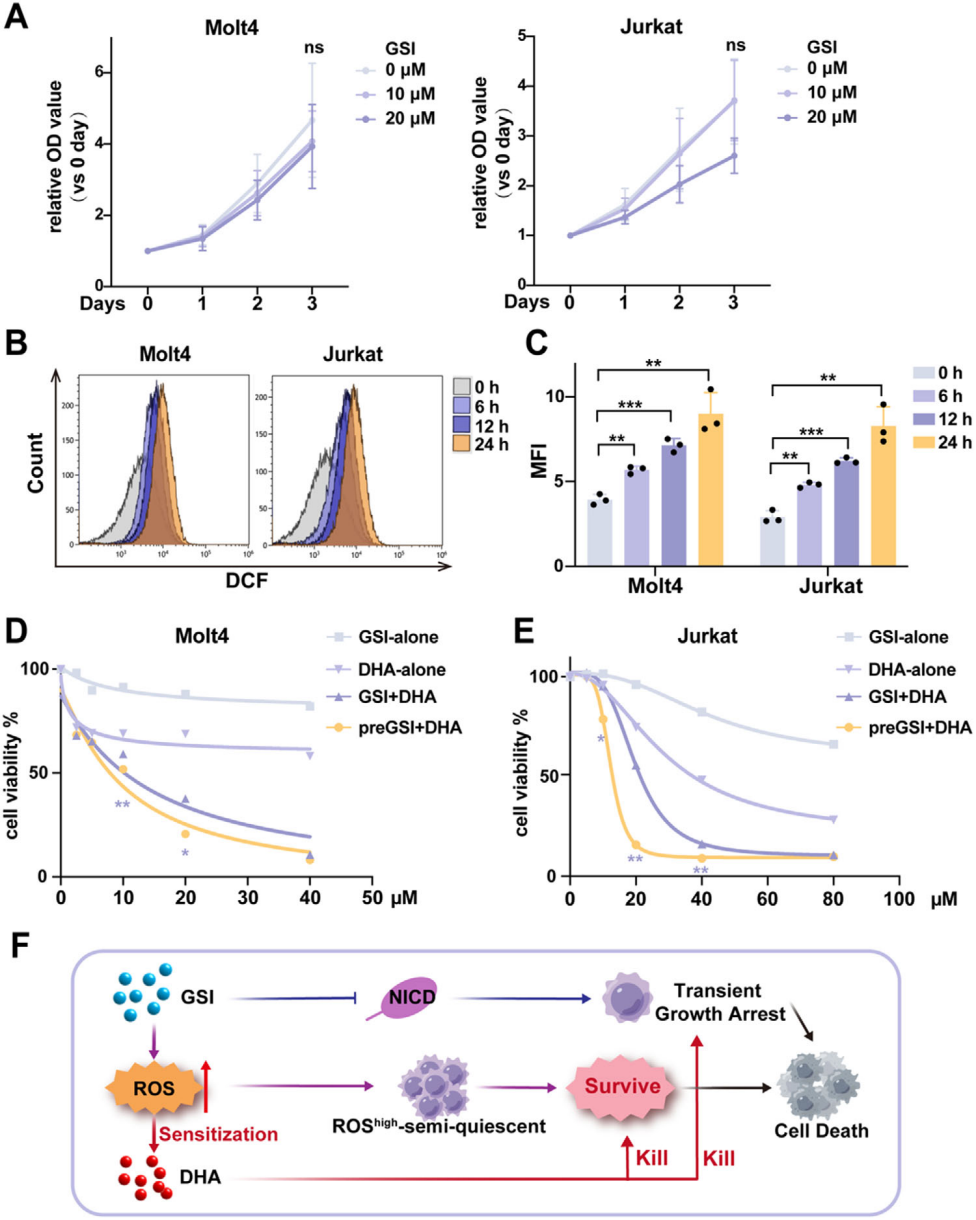
Follow‐up treatment with DHA enhances GSI cytotoxicity. A) Proliferative activity of T‐ALL cells treated with different concentrations of GSI (*n* = 3 independent experiments). ns, not significant versus 10 or 20 µm concentrations. B) Flow cytometric analysis of ROS levels in T‐ALL cells following treatment with GSI (20 µm) at different time points. C) Quantification of mean fluorescence intensity (MFI) for ROS detection as shown in (B) (*n* = 3 independent experiments). D,E) Cell viability (CCK8 assay) of Molt4 (D) and Jurkat (E) cells after different treatments: GSI alone for 24 h, DHA alone for 24 h, GSI+DHA for 24 h, pre‐treatment with GSI for 24 h followed by co‐treatment with DHA for 24 h (*n* = 3 independent experiments). *, versus The GSI+DHA group. F) Scheme of synergistic interaction between DHA and GSI. Data represent mean ± s.d. Two‐tailed Student's *t*‐tests were used to assess statistical significance. *p < 0.05, **p < 0.01, ***p < 0.001.

When the proliferation‐inhibiting effect of GSI was evaluated in T‐ALL cell lines, no significant inhibition in cell proliferation was observed over time following GSI treatment (**Figure** **1**A). These results suggest that GSI in solution has limited cytotoxicity against T‐ALL cells, likely due to its low aqueous solubility, which is consistent with previous reports.^[^ ^26^ ^]^ To confirm the temporal characteristics of ROS elevation^[^ ^27^, ^28^ ^]^ and guide the optimal window for DHA addition, we monitored intracellular ROS levels in GSI‐treated Molt4 and Jurkat cells using DCFH‐DA (Figure 1B). ROS levels gradually increased throughout the treatment period (Figure 1C).

We next assessed the cytotoxic effects of combining GSI with DHA in T‐ALL cells. At the maximal tested concentrations of GSI, the mean cellular inhibition rates were quantified at 17.9% in Molt4 and 33.9% in Jurkat. Treatment of DHA with the maximum concentration resulted in a cellular inhibition rate of 41.8% in Molt4, and the IC50 of DHA was 28.78 µm in Jurkat. However, co‐treatment with DHA significantly enhanced GSI cytotoxicity. When combined in a 1:1 molar ratio, the IC50 of GSI decreased to 14.2 µm in Molt4 and 19.83 µm in Jurkat (Figure 1D,E), indicating that DHA potentiates the cytotoxic effect of GSI. Importantly, when T‐ALL cells were pre‐incubated with GSI followed by DHA co‐treatment, cell viability was further reduced, with IC50 values dropping to 10.07 µm in Molt4 and 12.36 µm in Jurkat. These findings suggest that GSI pre‐treatment is more effective at sensitizing T‐ALL cells to DHA than simultaneous administration. We propose that GSI inhibits Notch1 activation and induces transient growth arrest in T‐ALL cells, during which a subpopulation of ROS^high^ semi‐quiescent cells persists due to increasing ROS levels following GSI exposure. This elevated ROS environment, in turn, sensitizes the cells to DHA, enhancing its cytotoxic effect (Figure 1F).

### 2.2 Successful Synthesis of pDMA‐pEPEMA‐pHD

The synthetic route for the ROS‐responsive polymer pDMA‐pEPEMA‐pHD is illustrated in Figure S1 (Supporting Information). First, the ROS‐responsive monomer HD was synthesized by linking HEA and DHA through a ROS‐sensitive bridge. The successful synthesis of HD was confirmed by ^1^H‐NMR spectroscopy. As shown in Figure S2A (Supporting Information), the ^1^H‐NMR results verify the successful formation of the HD monomer. To enhance the hydrophobicity of the polymer after polymerization, EPEMA was included in the structure. The EPEMA monomer was synthesized via a two‐step substitution reaction. First, the intermediate EPEA was prepared through a substitution reaction between N‐ethyl ethanolamine and bromopropane. The successful synthesis of EPEA was confirmed by ^1^H‐NMR analysis (Figure S2B, Supporting Information). Next, EPEMA was obtained by a subsequent substitution reaction between EPEA and methacryloyl chloride. The resulting product was confirmed as EPEMA by ^1^H‐NMR, as shown in Figure S2C (Supporting Information).

In this study, pDMA was selected as the hydrophilic block of the polymer. Accordingly, pDMA was first synthesized via RAFT polymerization using DCT as the CTA and AIBN as the initiator. As shown in Figure S2D (Supporting Information), the ^1^H‐NMR results confirmed the successful polymerization of pDMA. Subsequently, the block copolymer pDMA–pEPEMA was synthesized using the same RAFT polymerization method. The ^1^H‐NMR spectrum confirmed the successful formation of pDMA‐pEPEMA (Figure S2E, Supporting Information). Finally, the ROS‐responsive monomer HD was polymerized onto the pre‐synthesized two‐block copolymer pDMA‐pEPEMA, yielding the final three‐block ROS‐responsive polymer pDMA–pEPEMA–pHD. This triblock copolymer enables both nanoparticle formation and ROS‐triggered release of DHA. As shown in Figure S2F (Supporting Information), the successful synthesis of pDMA‐pEPEMA‐pHD was confirmed by ^1^H‐NMR. Comparison of the ^1^H‐NMR spectra of pDMA‐pEPEMA‐pHD before (T0) and after (TN) polymerization indicated a monomer conversion rate of HD of 63.74%.

### 2.3 Successful Preparation of ROS‐Responsive NPs

**Figure 2:**
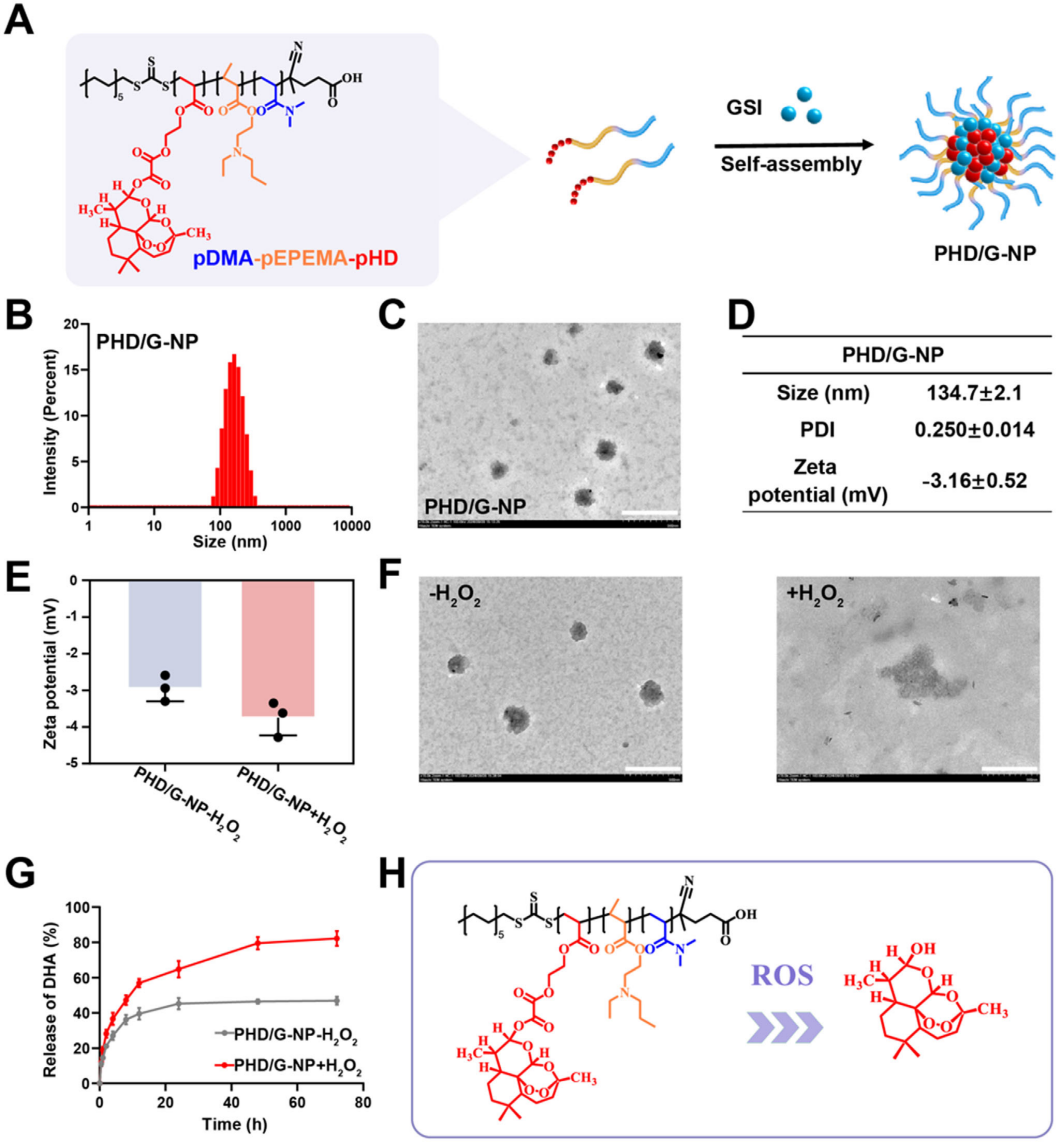
Preparation and characterization of PHD/G‐NPs and CD38‐PHD/G‐NPs. A) Schematic diagram illustrating the preparation of PHD/G‐NPs. B) Particle size distribution of PHD/G‐NPs evaluated by DLS. C) Representative TEM image of PHD/G‐NPs. Scale bar = 500 nm. D) Particle size, PDI, and zeta potential values of PHD/G‐NPs. E) Zeta potential changes of PHD/G‐NPs after incubation in pH 7.4 PBS (with or without 1 mm H_2_O_2_) (*n* = 3 independent experiments). F) TEM images of PHD/G‐NPs after incubation in pH 7.4 PBS (with or without 1 mm H_2_O_2_). Scale bar = 500 nm. G) In vitro release profile of DHA from PHD/G‐NPs after incubation in pH 7.4 PBS (with or without 1 mm H_2_O_2_). H) Schematic diagram of ROS‐responsive DHA release from PHD/G‐NPs. Data represent mean ± s.d.

Based on the ROS‐responsiveness of the pDMA‐pEPEMA‐pHD polymer, ROS‐responsive NPs were successfully prepared using the nanoprecipitation method, as illustrated in **Figure** **2**A. The particle size, PDI, and zeta potential of the resulting PHD/G‐NPs were measured using DLS. As shown in Figure 2B,D,E, the average particle size was 134.7 ± 2.1 nm, with a uniform distribution (PDI: 0.25 ± 0.014). The zeta potential was measured at −3.16 ± 0.52 (Figure 2D). Transmission electron microscopy (TEM) was used to examine the morphology of the nanoparticles. As shown in Figure 2C, the PHD/G‐NPs were spherical and well‐dispersed, confirming successful nanoparticle formation.

To evaluate ROS‐responsiveness, changes in nanoparticle morphology and zeta potential were assessed after incubation in phosphate‐buffered saline (PBS, pH 7.4) containing 1 mm H_2_O_2_. As shown in Figure 2E,F, the nanoparticle structure was disrupted and the zeta potential altered, indicating responsiveness to ROS. Finally, the in vitro release profile of DHA was studied using a dynamic membrane dialysis method. As shown in Figure 2G, the cumulative release of DHA reached 82.16 ± 3.43% in PBS (pH 7.4) containing 1 mm H_2_O_2_, compared to 46.94 ± 1.9% in PBS without H_2_O_2_. These results demonstrate the ROS‐triggered release behavior of DHA from the nanoparticles (Figure 2H).

### 2.4 Anti‐Leukemia Effects of PHD/G‐NPs in vitro

**Figure 3:**
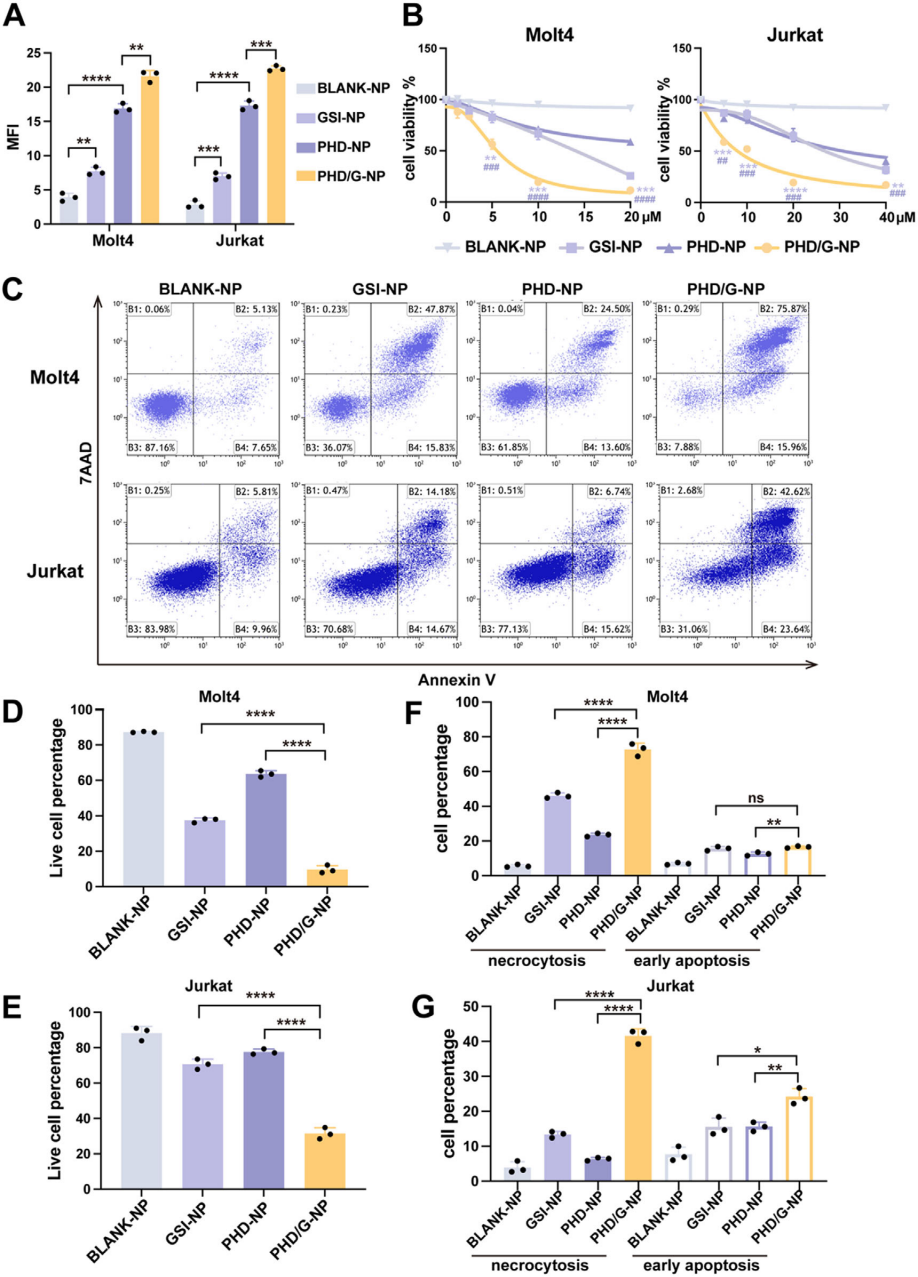
Effects of PHD/G‐NPs on inducing cell death in vitro. A) Quantification of MFI for ROS detection by flow cytometric analysis (*n* = 3 independent experiments). B) Cell viability of Molt4 (left) and Jurkat (right) cells treated with different nanoparticles measured by CCK8 assay (*n *= 3 independent experiments). *, versus The GSI‐NP group. #, versus The PHD‐NP group. C) Representative cell death analysis of T‐ALL cell lines after treatment with various formulations (20 µm). D,E) Statistical histograms showing the live cell percentages (*n* = 3 independent experiments) for Molt4 (D) and Jurkat (E) cells from (C). F,G) Statistical histograms showing the percentages of necrosis and early apoptosis in Molt4 (F) and Jurkat (G) cells from (C). Data represent mean ± s.d. Two‐tailed Student's *t*‐tests were used to assess statistical significance. ns. *p* > 0.05, *p < 0.05, **p < 0.01, ***p < 0.001, ****p < 0.0001.

We quantified the intracellular levels of ROS under the treatment of nanoparticles for 6 hours with a concentration of 10 µm. The results demonstrated that GSI‐NPs exhibited a significant elevation in ROS level compared to BLANK‐NPs, paralleling the efficacy in free GSI. PHD‐NPs improved a higher ROS level than BLANK‐NPs, in consistent with the mechanisms of DHA‐driven oxidative stress potentiation.^[^ ^29^ ^]^ Due to the enhanced effect of DHA by GSI‐mediated ROS elevation, the synergistic interaction between DHA and GSI resulted in the highest ROS level observed in the PHD/G‐NPs group (**Figure** **3**A). We next evaluated the anti‐leukemic efficacy of PHD/G‐NPs against T‐ALL cells in vitro using the CCK8 assay and flow cytometry. The results demonstrated that GSI‐NPs significantly enhanced cytotoxicity against two T‐ALL cell lines compared to free GSI. However, both GSI‐NPs and PHD‐NPs exhibited weaker inhibitory effects than PHD/G‐NPs, with statistically significant differences (Figure 3B). The IC50 values of PHD/G‐NPs were 5.6 µm in Molt4 cells and 8.6 µm in Jurkat cells (Figure 3B). Treatment with 20 µm PHD/G‐NPs resulted in the lowest cell viability in both T‐ALL cell lines, as determined by apoptosis assays (Figure 3C–E). Additionally, PHD/G‐NPs induced a stronger effect on necrotic cell death rather than early apoptosis (Figure 3F,G), suggesting that DHA may exert its cytotoxicity primarily through non‐apoptotic mechanisms of cell death.

### 2.5 Mechanism of PHD/G‐NPs‐Induced Facilitated Ferroptosis

**Figure 4:**
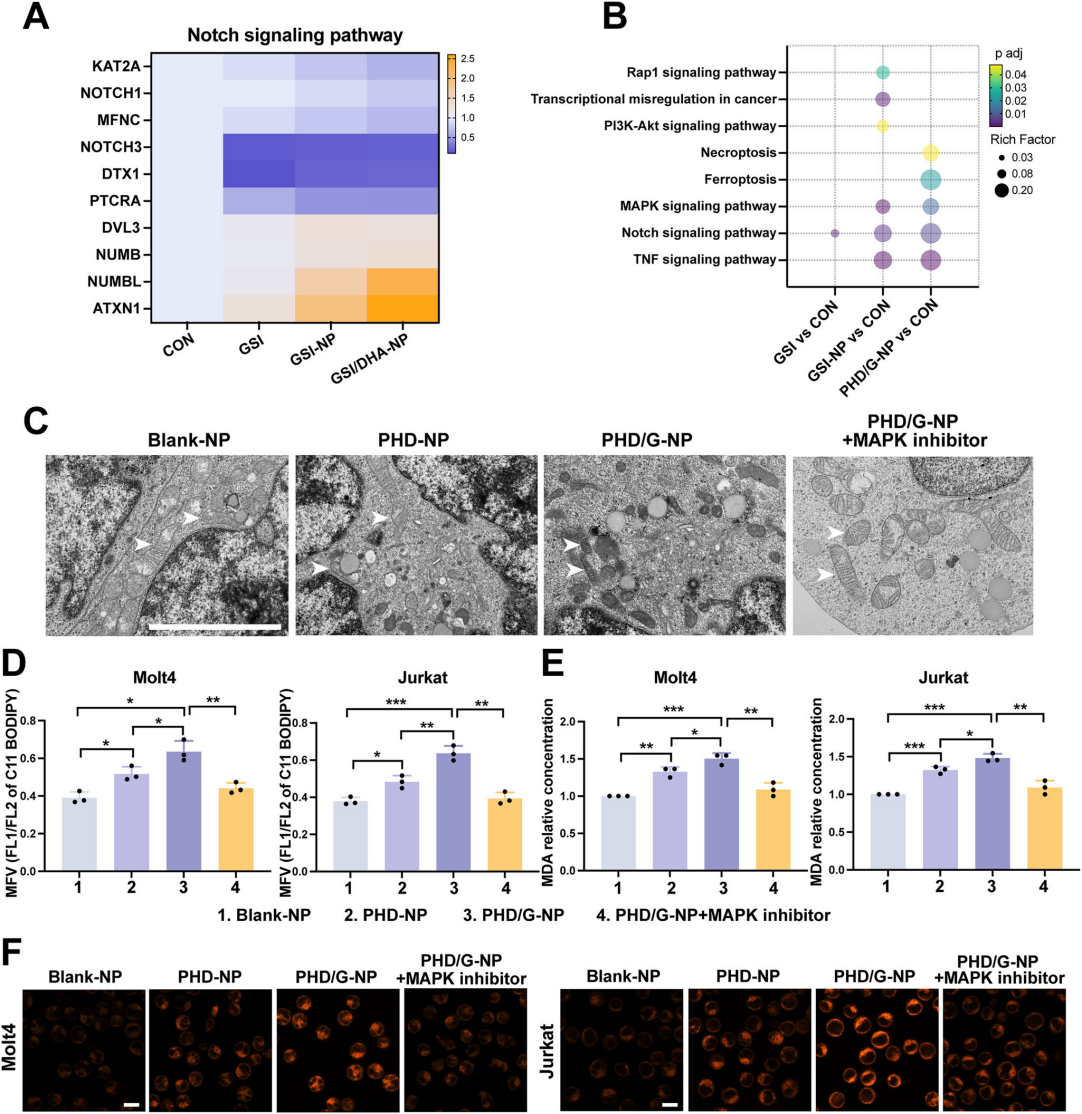
PHD/G‐NPs induced facilitated ferroptosis by activating MAPK. A) Heatmap showing the relative expression of genes enriched in the Notch signaling pathway. B) KEGG enrichment analysis of differentially expressed genes (DEGs) compared with the control group. C) Representative TEM images of Molt4 cells after different treatments showing the degree of ferroptosis. Scale bar = 500 nm. D) Mean fluorescence value (MFV) analysis of C11 BODIPY staining in Molt4 (left) and Jurkat (right) cells following different treatments, assessed by flow cytometry (*n* = 3 independent experiments). E) Statistical histograms showing relative MDA levels in Molt4 (left) and Jurkat (right) cells after different treatments (*n* = 3 independent experiments). F) Immunofluorescence analysis of Fe^2+^ in Molt4 (left) and Jurkat (right) after the different administrations. Scale bar = 12.5 µm. Data represent mean ± s.d. Two‐tailed Student's *t*‐tests were used to assess statistical significance. *p < 0.05, **p < 0.01, ***p < 0.001.

To elucidate the mechanism of cell death induced by PHD/G‐NPs, RNA‐Seq was conducted to systematically characterize gene expression differences in Molt4 cells treated with PBS, GSI, GSI‐NPs, or PHD/G‐NPs. Genes associated with the Notch signaling pathway were analyzed, and their expression profiles are shown in the heatmap in **Figure** **4**A. Compared to the control group (treated with PBS), the Notch‐positive regulatory genes (KAT2A, NOTCH1, MFNC, NOTCH3, DTX1, PTCRA) were downregulated in the GSI, GSI‐NPs, and PHD/G‐NPs groups. Conversely, Notch‐negative regulatory genes (DVL3, NUMB, NUMBL, ATXN1) were upregulated (Figure 4A), indicating that the GSI component effectively inhibited Notch1 activation. Next, we performed Kyoto Encyclopedia of Genes and Genomes (KEGG) pathway enrichment analysis on the differentially expressed genes identified in GSI, GSI‐NPs, and PHD/G‐NPs treatment groups compared to the control. The results revealed activation of the PI3K‐AKT signaling pathway in the GSI‐NPs group, a pathway associated with downstream effects of elevated ROS levels.^[^ ^30^ ^]^ Additionally, the MAPK signaling pathway was enriched in both GSI‐NPs and PHD/G‐NPs groups, while ferroptosis‐related genes were specifically enriched in the PHD/G‐NPs group (Figure 4B). These findings suggest that DHA enhances GSI‐induced cytotoxicity by promoting ferroptosis, and that GSI, in turn, sensitizes cells to DHA‐induced ferroptosis by activating the MAPK signaling pathway—a known regulator of ferroptosis.^[^ ^31^ ^]^

To further validate our hypothesis, we treated T‐ALL cells in the PHD/G‐NPs group with a MAPK inhibitor (SB202190, an inhibitor of the p38 MAPK signaling pathway). Typical ultrastructural features of ferroptosis—including reduced mitochondrial size, increased membrane density, and loss of cristae—were observed by TEM following treatment with PHD‐NPs. These morphological changes were significantly more pronounced in cells treated with PHD/G‐NPs. However, treatment with the MAPK inhibitor largely restored mitochondrial morphology to a normal state (Figure 4C). Previous studies have shown that ferroptosis is marked by increased lipid ROS generation and elevated mitochondrial malondialdehyde (MDA) levels.^[^ ^32^, ^33^ ^]^ Consistent with these findings, PHD/G‐NPs significantly increased lipid ROS levels, as indicated by BODIPY‐C11 staining (Figure 4D; Figure S3, Supporting Information), as well as MDA levels, a product of lipid peroxidation (Figure 4E).^[^ ^34^ ^]^ Notably, both lipid ROS and MDA levels were markedly reduced upon MAPK inhibition. Additionally, immunofluorescence analysis of Fe^2+^ levels yielded consistent results, further supporting the role of MAPK signaling in ferroptosis induction (Figure 4F). Collectively, these findings confirm that GSI enhances DHA‐induced ferroptosis by activating the MAPK signaling pathway, thereby compensating for its own limited cytotoxicity.

### 2.6 Anti‐Leukemia Effects of PHD/G‐NPs in vivo

**Figure 5:**
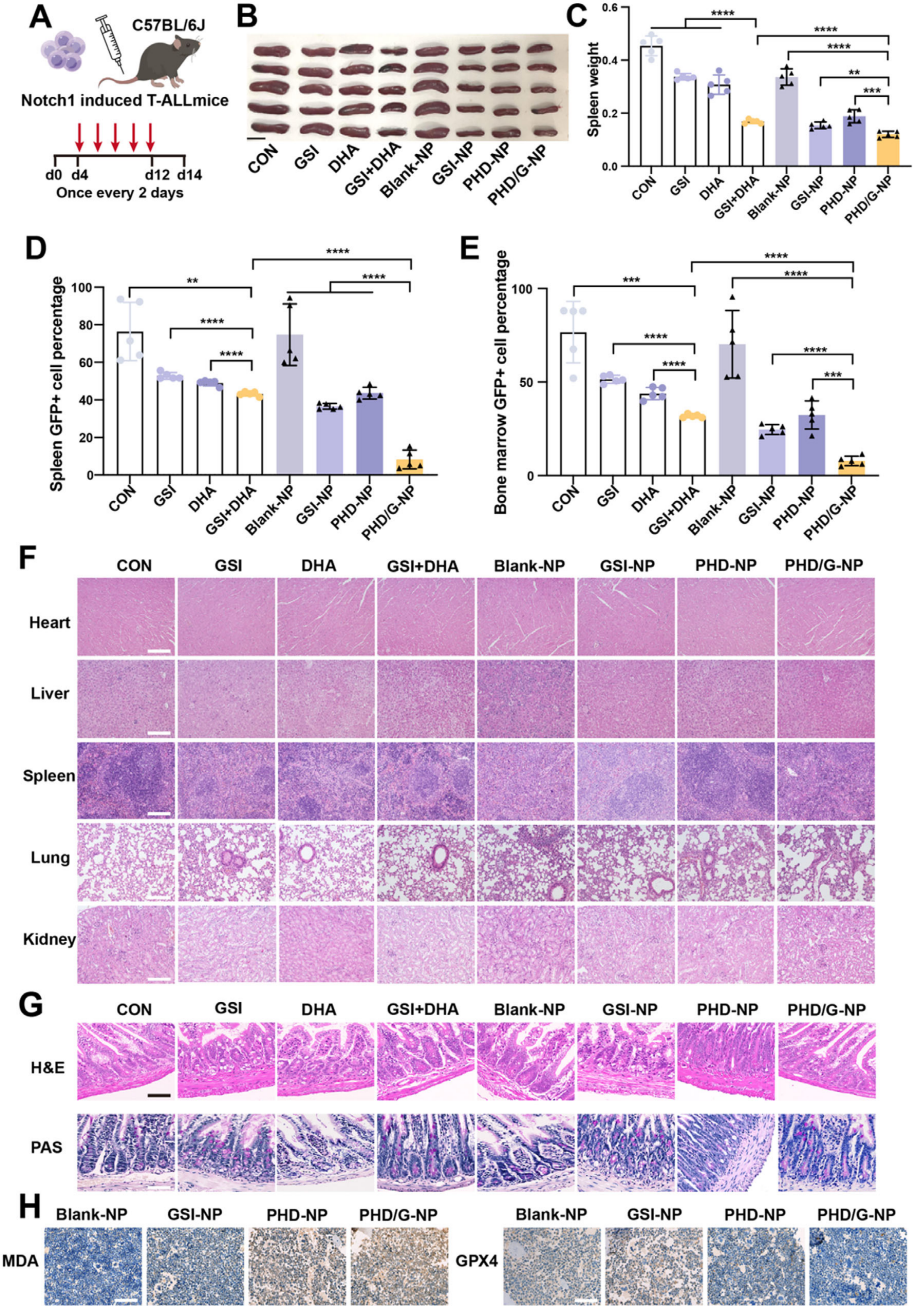
Effects of PHD/G‐NPs on anti‐leukemia activity and ferroptosis in vivo. A) Experimental schedule for in vivo antileukemic studies in Notch1‐induced T‐ALL mice. B,C) Photographs showing spleen size (B) and corresponding statistical histograms of spleen weight (C) across eight treatment groups. Scale bar = 1 cm. D) Flow cytometric analysis of GFP+ T‐ALL cell percentages in spleen samples. E) Flow cytometric analysis of GFP+ cell percentages in bone marrow samples. F) Microscopy images of H&E‐stained sections of major organs following different treatments. Scale bar = 150 µm. G) Microscopy images of H&E and PAS staining of the ileum at the end of treatment. Scale bar = 75 µm. H) Immunohistochemical analysis of MDA (left) and GPX4 (right) expression levels in femur tissue following different treatments. Scale bar = 75 µm. *n* = 5 mice per group. Data represent mean ± s.d. Two‐tailed Student's *t*‐tests were used to assess statistical significance. *p < 0.05, **p < 0.01, ***p < 0.001, ****p < 0.0001.

We evaluated the anti‐leukemic efficacy of the nanoparticles in a Notch1‐induced T‐ALL mouse model. Free drugs or nanoparticle formulations were administered via tail vein injection five times (GSI, 30 mg kg^−1^; Molar concentration of GSI: DHA = 1:1), as illustrated in **Figure** **5**A. Treatment with the combination of free GSI and DHA significantly reduced both spleen size and weight compared with either GSI or DHA alone. Notably, PHD/G‐NPs more effectively suppressed splenomegaly than either GSI‐NPs or PHD‐NPs (Figure 5B,C). In line with these results, the percentage of GFP+ leukemic cells in both the spleen and bone marrow (BM) was significantly reduced following PHD/G‐NPs treatment (Figure 5D,E). Encouragingly, PHD/G‐NPs demonstrated the highest anti‐leukemic efficacy among all treatment groups.

To assess the biosafety of the nanoparticles, we further examined the histological morphology of major organs in Notch1‐induced T‐ALL mice using H&E staining. Histopathological analysis of the heart, liver, spleen, lungs, and kidneys revealed no observable abnormalities or pathological changes compared with the control group (Figure 5F). These results indicate that PHD/G‐NPs are safe for intravenous administration in vivo.

To assess the effect of PHD/G‐NPs on GSI‐induced gastrointestinal toxicity, ileum sections from T‐ALL mice treated with various formulations of free drugs and nanoparticles were analyzed and compared to those from the control group. H&E and periodic acid‐schiff (PAS) staining of the ileum revealed that the GSI component induced visible intestinal secretory metaplasia, characterized by a marked increase in the number of goblet cells (Figure 5G). All mice treated with GSI, GSI+DHA, GSI‐NPs, and PHD/G‐NPs exhibited elevated goblet cell counts, indicating that PHD/G‐NPs require a targeted delivery mechanism to selectively direct treatment toward T‐ALL cells and minimize off‐target effects.

We next evaluated the ferroptosis‐inducing potential of PHD/G‐NPs in Notch1‐induced T‐ALL mice. Immunohistochemical analysis of MDA and GPX4, biomarkers of ferroptosis,^[^ ^35^ ^]^ in bone marrow biopsies revealed significant ferroptotic activity in mice treated with PHD‐NPs and PHD/G‐NPs, with the latter demonstrating the highest levels of ferroptosis among all groups (Figure 5H). These findings suggest that PHD/G‐NPs possess potent anti‐leukemic activity and a strong ferroptosis‐inducing effect with acceptable in vivo safety; however, they do not prevent GSI‐associated intestinal epithelial damage.

### 2.7 PHD/G‐NPs Synergistically Activate Anti‐Tumor Immune Responses Combined with αPD‐1 in vivo

**Figure 6:**
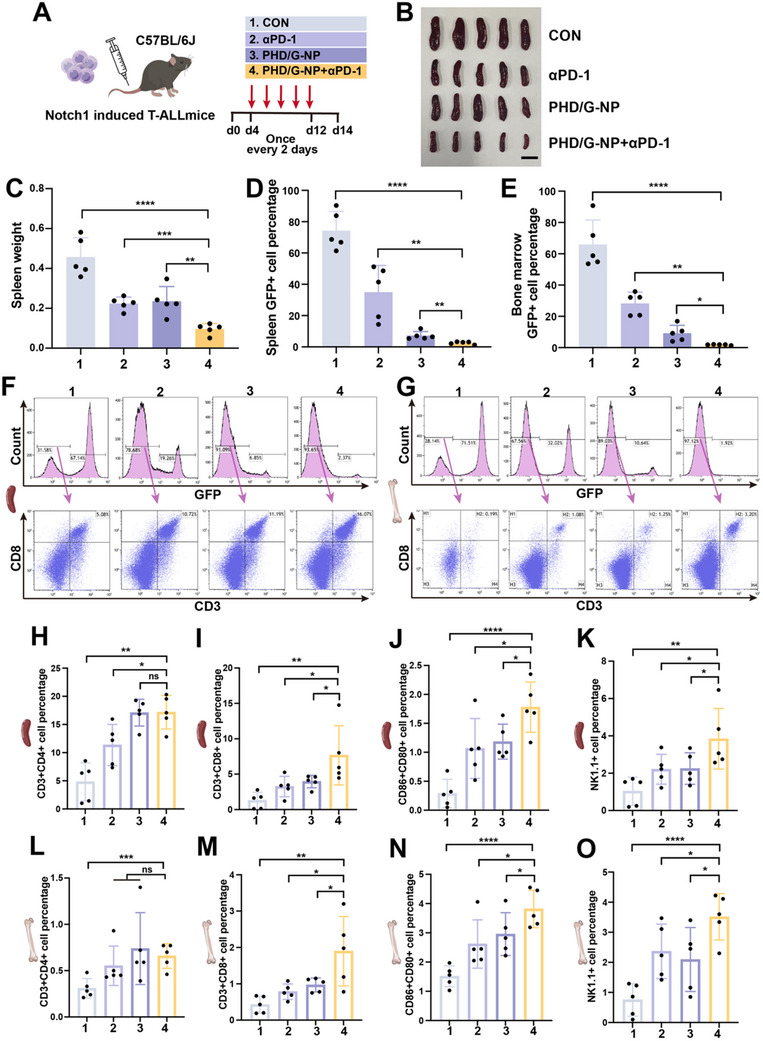
Anti‐tumor immune responses of PHD/G‐NPs combined with αPD‐1 in vivo. A) Experimental schedule for different treatments in Notch1‐induced T‐ALL mice: 1, PBS (CON); 2, αPD‐1; 3, PHD/G‐NP; 4, PHD/G‐NP combined with αPD‐1. B,C) Photographs showing spleen size (B) and corresponding statistical histograms of spleen weight (C) across the four groups. Scale bar = 1 cm. D) Flow cytometric analysis of GFP+ T‐ALL cell percentages in spleens. E) Flow cytometric analysis of the GFP+ cell percentages in bone marrow samples. F,G) Flow cytometric analysis of the CD3+CD8+ T cell percentages among GFP‐ cells in spleen (F) and bone marrow (G). H–K) Statistical histograms showing percentages of CD3+CD4+ T cells (H), CD3+CD8+ T cells (I), CD86+CD80+ DCs (J), and NK1.1+ NK cells (K) among GFP‐ cells in spleen. L–O) Statistical histograms showing percentages of CD3+CD4+ T cells (L), CD3+CD8+ T cells (M), CD86+CD80+ DCs (N), and NK1.1+ NK cells (O) among GFP‐ cells in bone marrow. *n *= 5 mice per group. Data represent mean ± s.d. Two‐tailed Student's *t*‐tests were used to assess statistical significance. *p < 0.05, **p < 0.01, ***p < 0.001, ****p < 0.0001.

Xu et al. previously reported that PD‐1 expression is positively correlated with the malignant progression of T‐ALL, and that PD‐1 blockade can significantly eradicate T‐ALL stem cells and suppress tumor progression.^[^ ^36^ ^]^ Additionally, DAMPs released during ferroptosis can reprogram the tumor microenvironment, and our earlier results demonstrated that PHD/G‐NPs effectively induce ferroptosis. Therefore, we hypothesized that combining PHD/G‐NPs with PD‐1 antibody (αPD‐1) would synergistically enhance anti‐tumor efficacy. To test this, a Notch1‐induced T‐ALL mouse model was established to evaluate the leukemia‐suppressive and immune‐activating effects of different treatments. Mice were intravenously administered PBS, αPD‐1, PHD/G‐NPs, or the combination of PHD/G‐NPs+αPD‐1 five times (**Figure** **6**A). The combination therapy significantly reduced splenomegaly and the percentage of GFP+ leukemic cells in both spleen and BM compared to the PBS, αPD‐1, and PHD/G‐NPs groups (Figure 6B–E).

To further assess the immune responses activated by the treatments, we analyzed T cell activation in bone marrow and spleen via flow cytometry. The percentage of CD8+ T cells among GFP‐ cells in both bone marrow and spleen was significantly higher in the PHD/G‐NPs+αPD‐1 group than in any other groups (Figure 6F,G,I,M). In contrast, CD4+ T cell levels showed no significant differences among the groups (Figure 6H,L; Figure S4, Supporting Information). Moreover, PHD/G‐NPs+αPD‐1 treatment enhanced DC maturation in vivo, as evidenced by a higher percentage of mature DCs in GFP‐cells compared to other groups (Figure 6J,N; Figure S4, Supporting Information). In addition, NK cell accumulation in both bone marrow and spleen was increased under PHD/G‐NPs+αPD‐1 (Figure 6K,O; Figure S4, Supporting Information).

### 2.8 Comprehensive Effects of CD38‐PHD/G‐NPs in T‐ALL CDX Model

**Figure 7:**
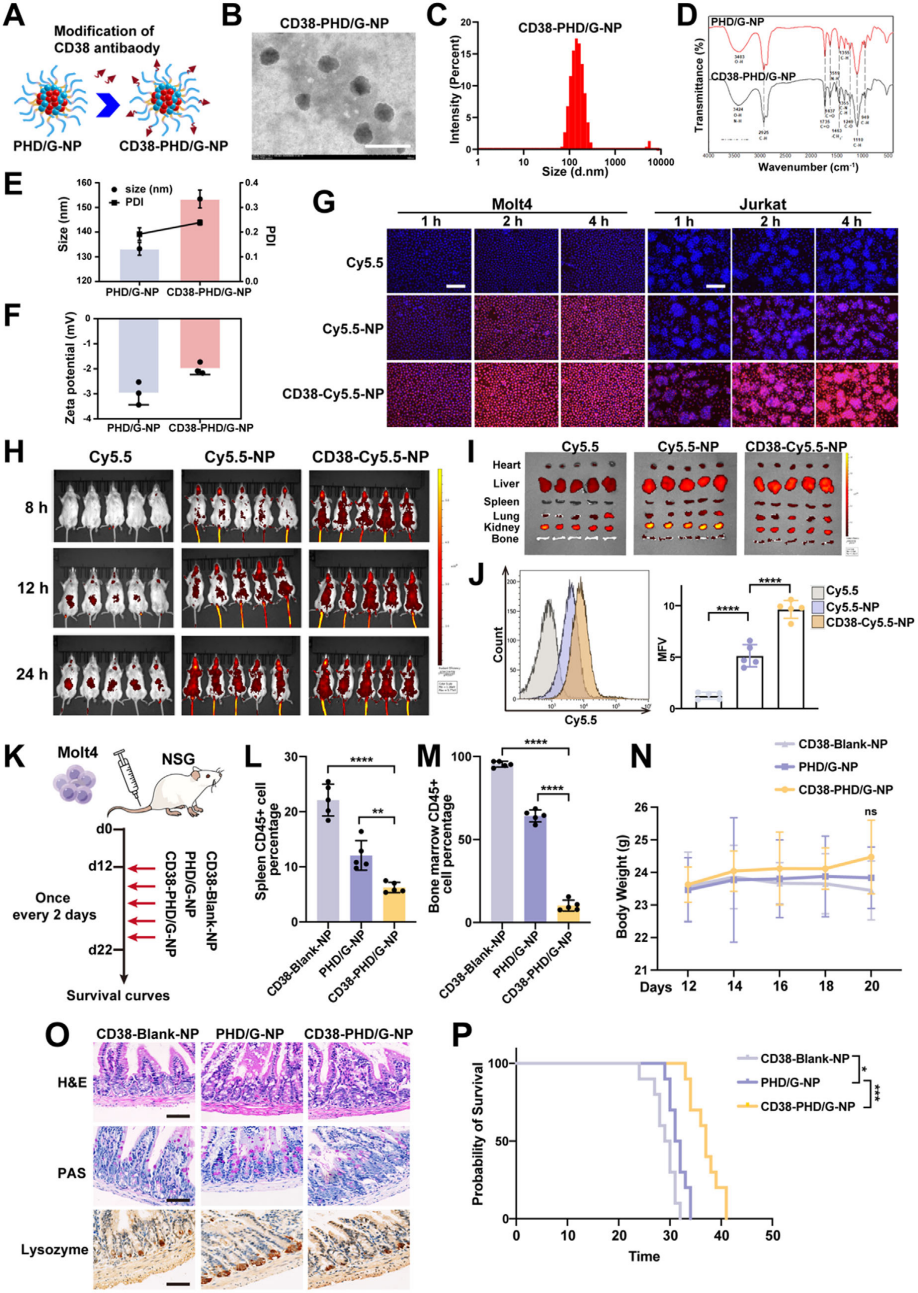
Targeted delivery and effects of CD38‐PHD/G‐NPs in T‐ALL CDX model. A) Schematic diagram illustrating the preparation of CD38‐PHD/G‐NPs. B) Representative TEM image of CD38‐PHD/G‐NPs. Scale bar = 500 nm. C) Particle size distribution of CD38‐PHD/G‐NPs evaluated by DLS. D) FTIR spectra of PHD/G‐NPs and CD38‐PHD/G‐NPs. E,F) Comparison of particle size and PDI E) and zeta potential F) between PHD/G‐NPs and CD38‐PHD/G‐NPs measured by DLS (*n* = 3 independent experiments). G) Fluorescence images of T‐ALL cell lines incubated with free Cy5.5, Cy5.5‐NPs, and CD38‐Cy5.5‐NPs for 1, 2, or 4 h. Red indicates Cy5.5; blue indicates nuclei. Scale bar = 75 µm. H) in vivo fluorescence imaging of Molt4‐bearing mice treated with free Cy5.5, Cy5.5‐NPs, or CD38‐Cy5.5‐NPs at different time points. I) *Ex vivo* fluorescence imaging of femurs and major organs from Molt4‐bearing mice 24 h post‐administration. J) Flow cytometric analysis of Cy5.5 uptake in bone marrow cells from Molt4‐bearing mice at 24 h post‐administration. K) Schematic diagram of the xenotransplantation experiment. L) Flow cytometric analysis of human CD45+ T‐ALL cell percentages in spleens. M) Flow cytometric analysis of human CD45+ cell percentages in bone marrow. N) Body weight changes of mice treated with different nanoparticle formulations. O) Microscopy images of H&E staining, PAS staining, and immunohistochemical analysis of lysozyme expression in the ileum following different treatments. Scale bar = 75 µm. P) Kaplan–Meier survival curves of mice treated with different nanoparticle formulations. *n* = 5 mice per group. Data represent mean ± s.d. Two‐tailed Student's *t‐*tests were used to assess statistical significance. Survival analysis was performed using Kaplan–Meier survival plots and log‐rank tests. *p < 0.05, **p < 0.01, ***p < 0.001, ****p < 0.0001.

To mitigate the severe gastrointestinal side effects associated with GSI, we modified PHD/G‐NPs by conjugating them with a CD38 antibody (**Figure** **7**A). The morphology of the resulting NPs was examined using TEM. As shown in Figure 7B, CD38‐PHD/G‐NPs were spherical and well‐dispersed. The particle size, PDI, and zeta potential of CD38‐PHD/G‐NPs were measured by DLS. The average particle size was 153.4 ± 2.9 nm (Figure 7C, E), and with a uniform distribution (PDI: 0.238 ± 0.008) (Figure 7E). The zeta potential was −1.99 ± 0.19 mV (Figure 7F). The differences in particle size and zeta potential between CD38‐PHD/G‐NPs and unmodified PHD/G‐NPs provide preliminary evidence of successful CD38 antibody conjugation. To further confirm the modification, infrared spectroscopy analysis was performed. As shown in Figure 7D, a new peak appeared at 1519 cm^−1^ in the spectrum of CD38‐PHD/G‐NPs, attributed to the N–H bending vibration (amide II band) of the CD38 antibody. This confirmed the successful surface modification of the nanoparticles with the CD38 antibody.

To evaluate whether CD38 antibody modification enhances cellular uptake by T‐ALL cells in vitro, we used the fluorescent dye Cy5.5 to track uptake efficiency. Fluorescence microscopy and flow cytometry analyses revealed minimal uptake of free Cy5.5 by T‐ALL cells during the observation period. In contrast, cells treated with CD38‐Cy5.5‐NPs exhibited significantly stronger fluorescence than those treated with Cy5.5‐NPs, indicating that CD38 conjugation actively promoted targeted uptake by T‐ALL cells (Figure 7G). Flow cytometry results supported this finding (Figure S5A, Supporting Information). To assess in vivo distribution, we monitored the uptake of CD38‐Cy5.5‐NPs using bioluminescence imaging in T‐ALL mice. The results showed that CD38‐Cy5.5‐NPs had a substantially higher uptake rate compared to other formulations (Figure 7H; Figure S5B, Supporting Information), confirming their enhanced targeting efficiency in vivo.

More importantly, an *ex vivo* imaging study of major organs and bone marrow was conducted 24 h after nanoparticle administration. CD38‐Cy5.5‐NPs were more prominently internalized in the bone marrow and spleen—sites with high leukemic cell abundance—in T‐ALL mice (Figure 7I; Figure S5C, Supporting Information). Furthermore, flow cytometry analysis showed that the MFI of bone marrow cells in the CD38‐Cy5.5‐NPs group was significantly higher than in the other groups (Figure 7J). These findings suggest that CD38‐Cy5.5‐NPs preferentially target leukemic cells in vivo.

To evaluate the leukemia‐suppressive effects of CD38‐Cy5.5‐NPs in vivo, NSG mice were engrafted with Molt4 cells and treated with CD38‐Blank‐NPs, PHD/G‐NPs, or CD38‐PHD/G‐NPs. All treatments were administered intravenously five times, as illustrated in Figure 7K. The results showed that CD38‐PHD/G‐NPs most effectively suppressed splenomegaly and reduced the percentage of GFP+ leukemic cells in both the spleen and bone marrow, compared with CD38‐Blank‐NPs and PHD/G‐NPs (Figure 7L,M). No significant differences in body weight were observed among the three groups (Figure 7N). Remarkably, mice treated with CD38‐PHD/G‐NPs exhibited near‐normal goblet cell counts and preserved intestinal epithelial architecture (Figure 7O), indicating mitigation of GSI‐associated gastrointestinal side effects. Additionally, survival monitoring revealed that Molt4‐bearing mice treated with CD38‐PHD/G‐NPs had the longest survival compared to other groups (Figure 7P). These data confirm that CD38‐PHD/G‐NPs significantly enhance the combined therapeutic effects by both inhibiting leukemia progression and alleviating severe gastrointestinal toxicity. Due to the lack of anti‐tumor immune response in NSG mice, the efficacy of PHD/G‐NPs against leukemic burden is reduced in Molt4‐bearing mice compared with Notch1‐induced mice.

## 3 Conclusion

We have developed a self‐assembling nanotherapeutic strategy based on nanoparticle‐mediated co‐delivery of a GSI synergist and a ferroptosis sensitizer to overcome the challenges of targeted therapy in T‐ALL. In this study, PHD/G‐NPs were designed to release GSI first, followed by adaptive DHA release in response to elevated ROS levels. This controlled, sequential release strategy maximizes the synergistic anti‐leukemic efficacy of two agents with distinct mechanisms of action—simultaneously targeting Notch1‐mutated T‐ALL cells and compensating for each other's limitations. These findings offer a promising outlook for the development of convergent nanotherapeutic approaches to overcome current bottlenecks in T‐ALL targeted therapy.

## 4 Experimental Section

### Cell Lines and In Vitro Culture

The human T lymphoblastic cell lines Jurkat and Molt4 were purchased from the Chinese Academy of Sciences Cell Bank (Shanghai, China). Cells were cultured in RPMI‐1640 medium supplemented with 10% FBS and 1% penicillin–streptomycin at 37 °C in a humidified incubator containing 5% CO₂.

### Animals

Animal studies were conducted in compliance with institutional guidelines and were approved by the Medical Ethics Committee of Qilu Hospital of Shandong University (Approval NO. DWLL‐2023‐195). C57BL/6J mice (6–8 weeks old) were purchased from Beijing Vital River Laboratory Animal Technology. For the Notch1‐induced T‐ALL murine model, mouse pre‐leukemia cells infected with pMSCV‐NICD‐IRES‐GFP (GFP+Lin‐ cells) were intravenously injected into sublethally irradiated (7.5 Gy) C57BL/6J recipient mice. Notch1‐induced T‐ALL cells were labeled with GFP. Subsequently, GFP+ spleen cells (5 × 10⁵) from the second‐generation recipients were collected and intravenously injected into new recipients to establish the T‐ALL model. GFP+ leukemic cells in the BM and spleen were analyzed by flow cytometry. To establish the T‐ALL cell line‐derived xenograft (CDX) model, Molt4 cells (5 × 10^6^ cells per mouse) were transplanted into NSG mice (6–8 weeks old; GemPharmatech Laboratory). Human CD45+ leukemic cells in the BM and spleen were subsequently analyzed by flow cytometry.

### CCK8 Assay

T‐ALL cells were seeded into 96‐well plates at a density of 2 × 10^4^ cells per well. GSI and DHA were prepared at concentrations ranging from 0 to 80 µm and added to the wells. After incubation for predetermined time points, CCK8 reagent was added to each well and incubated for 3 h. The absorbance at 450 nm was measured using a microplate reader (BioTek, USA).

### Synthesis of pDMA‐pEPEMA‐pHD

The synthesis of pDMA‐pEPEMA‐pHD consists of two parts: monomer synthesis and polymer polymerization. The synthesis of monomers, including the synthesis of HEA‐DHA (HD) and EPEMA. The polymerization of polymers includes the polymerization of pDMA, pDMA‐pEPEMA, and pDMA‐pEPEMA‐pHD. The following is a detailed method for synthesis or polymerization.

### Synthesis of pDMA‐pEPEMA‐pHD—Synthesis of HD

Oxalyl chloride (3.65 mmol) was added to a round‐bottom flask and dissolved in anhydrous THF (5 mL). DHA (2.81 mmol) was separately dissolved in anhydrous THF (20 mL). At 0 °C, the DHA solution was slowly added dropwise into the oxalyl chloride solution, followed by the addition of triethylamine (3.93 mmol). The reaction mixture was stirred at 0 °C for 10 min, and then allowed to warm to room temperature and stirred for an additional 3 h. HEA (2.52 mmol) was dissolved in anhydrous THF. The reaction flask was cooled again to 0 °C, and the HEA solution was added dropwise, followed by triethylamine (7.59 mmol). The mixture was stirred at 0 °C for 10 min, and then at room temperature for 24 h. After completion of the reaction, the solvent was removed by rotary evaporation. The residue was dissolved in DCM and washed with Milli‐Q water. The organic layer was dried over anhydrous Na_2_SO_4_ and concentrated under reduced pressure. The crude product was purified by silica gel column chromatography to yield HD. The structure of HD was confirmed by ^1^H‐NMR.

N‐ethyl ethanolamine (0.15 mol), bromopropane (0.20 mol), and Na_2_CO_3_ (0.40 mol) were added to a round‐bottom flask and dissolved in acetonitrile. The reaction was conducted in an oil bath at 80 °C for 24 h. After completion, the solid Na_2_CO_3_ was removed by filtration, and the filtrate was concentrated by rotary evaporation. The residue was dissolved in ethyl acetate and extracted three times with saturated NaCl aqueous solution. The organic layer was dried over anhydrous Na_2_SO_4_, and the solvent was removed to obtain the intermediate EPEA. The structure of EPEA was confirmed by ^1^H‐NMR.

EPEA (12 mmol) and triethylamine (12 mmol) were added to a round‐bottom flask and dissolved in acetonitrile. At 0 °C, methacryloyl chloride (12 mmol) was added dropwise to the reaction mixture, which was then stirred at 0 °C for 2 h, and subsequently at room temperature for 24 h. Upon completion of the reaction, the mixture was filtered, and the filtrate was concentrated by rotary evaporation. The residue was dissolved in ethyl acetate and extracted three times with a saturated NaCl aqueous solution. After drying the organic layer over anhydrous Na_2_SO_4_, the solvent was evaporated to obtain the final product, EPEMA. The structure of EPEMA was confirmed by ^1^H‐NMR.

### Synthesis of pDMA‐pEPEMA‐pHD—Polymerization of pDMA

RAFT polymerization was selected for polymer preparation in this study. The detailed procedure for pDMA polymerization is as follows: DMA (20 mmol), 4‐cyano‐4‐(dodecylsulfanylthiocar‐bonyl) sulfanyl pentanoic acid (DCT) (0.4 mmol), 2,2′‐azobis(2‐methylpropionitrile) (AIBN) (0.08 mmol) and 1,4‐dioxane were added to a 25 mL Schlenk tube. After three freeze–thaw–vacuum cycles to remove oxygen, the mixture was polymerized at 80 °C for 4 h. The reaction solution was then precipitated three times using a mixture of diethyl ether and *n*‐hexane to yield pDMA. The successful synthesis of pDMA was confirmed by ^1^H‐NMR.

### Synthesis of pDMA‐pEPEMA‐pHD—Polymerization of pDMA‐pEPEMA

The procedure for the polymerization of pDMA–pEPEMA was as follows: pDMA (0.05 mmol), EPEMA (2.5 mmol), AIBN (0.015 mmol), and 1,4‐dioxane were added into a 25 mL Schlenk tube. After three freeze–thaw–vacuum cycles, the mixture was polymerized at 80 °C for 24 h. Following polymerization, the reaction solution was dialyzed against Milli‐Q water and freeze‐dried to obtain pDMA–pEPEMA. The successful polymerization of pDMA‐pEPEMA was verified by ^1^H‐NMR.

### Synthesis of pDMA‐pEPEMA‐pHD—Polymerization of pDMA‐pEPEMA‐pHD

pDMA–pEPEMA (0.0163 mmol), HD (0.3251 mmol), AIBN (0.0065 mmol), and DMF were added to a 25 mL Schlenk tube. After three freeze–thaw–vacuum cycles, the solution was polymerized at 80 °C for 24 h. Following polymerization, the reaction mixture was precipitated three times in diethyl ether to yield pDMA–pEPEMA–pHD. The successful polymerization of pDMA–pEPEMA–pHD was verified by ^1^H‐NMR.

### Preparation and Characterization of ROS Responsive Nanoparticles

Nanoparticles were prepared using the nanoprecipitation method. For the preparation of HD‐ and GSI‐co‐loaded nanoparticles (PHD/G‐NPs), pDMA‐pEPEMA‐pHD and GSI were dissolved in DMF and added dropwise into pH 7.4 PBS containing 1% Tween under sonication. Sonication was continued for an additional 30 min to complete nanoparticle formation. For the preparation of CD38 antibody‐modified nanoparticles (CD38‐PHD/G‐NPs), DMTMM was first dissolved in pH 7.4 PBS and added to the PHD/G‐NPs solution, followed by incubation at 37 °C for 30 min. Subsequently, the CD38 antibody was added to the mixture and incubated at 37 °C for 3 days to achieve antibody conjugation.

The particle size, PDI, and zeta potential of PHD/G‐NPs and CD38‐PHD/G‐NPs were measured using DLS (Malvern Instruments, UK). The morphology of the nanoparticles was observed by TEM (Hitachi, Japan). In addition, infrared spectroscopy analysis was conducted to verify the successful modification of the nanoparticles with the CD38 antibody.

Since ROS levels in T‐ALL could be increased by GSI treatment, a ROS‐responsive linkage was used in this study to bridge the polymer and DHA. Therefore, the ROS‐responsive properties of PHD/G‐NPs were evaluated. First, PHD/G‐NPs were prepared using the method described above. Then, the nanoparticles were incubated in pH 7.4 PBS (with or without 1 mm H_2_O_2_). Morphological changes and zeta potential were subsequently assessed by TEM and DLS to evaluate the ROS responsiveness of PHD/G‐NPs.

To further investigate the ROS‐responsive drug release behavior of DHA, release profiles were examined using dynamic membrane dialysis. The procedure was as follows: at a constant temperature of 37 °C, 1 mL of PHD/G‐NPs was placed into dialysis bags (molecular weight cutoff: 3500 Da) and incubated in 10 mL pH 7.4 PBS (containing 1% Tween, with or without 1 mm H_2_O_2_). At predetermined time points (0.5, 1, 2, 4, 8, 12, 24, 48, and 72 h), 1 mL of the release medium was collected and replaced with 1 mL of fresh medium. The amount of released DHA was quantified using HPLC.

### Flow Cytometry

For ROS detection, Reactive Oxygen Species Assay Kit (Yeasen Biotechnology, China) was used according to the manufacturer's instructions. For apoptosis assays, T‐ALL cells were stained with Annexin V/7‐AAD double staining (BestBio, China). The following antibodies were used for immunophenotyping: PerCP/Cy5.5 anti‐mouse CD3, APC anti‐mouse CD4, PE/Cy7 anti‐mouse CD8, PE anti‐mouse CD80, APC anti‐mouse CD86, PE anti‐mouse NK1.1, and APC anti‐human CD45 (all from BioLegend, San Diego, USA). Stained cells were analyzed by flow cytometry (Beckman Coulter), and the results were processed using Kaluza analysis software.

### Lipid Peroxidation Assay

The concentration of MDA was measured using a lipid peroxidation MDA assay kit (Beijing Solarbio Science & Technology) according to the manufacturer's instructions and detected with a microplate reader (BioTek, USA). For BODIPY‐C11 staining, cells were suspended in PBS containing 1 µm BODIPY C11 (581/591) (ABclonal) and incubated for 1 h at 37 °C. Cells were then washed twice with PBS. The fluorescence signal was detected by flow cytometry (Beckman Coulter) at FL1 (505–550 nm) and FL2 (>580 nm) channels and analyzed using Kaluza analysis software.

### Histology and Histochemistry

The heart, kidney, spleen, liver, lung, femur, and ileum were collected, fixed in 4% formaldehyde, and embedded in paraffin for sectioning. Hematoxylin and eosin (H&E) staining was performed following standard protocols. The ileum sections were additionally stained with PAS reagent. For histochemical analysis, anti‐MDA, anti‐GPX4, and anti‐Lysozyme antibodies were used as primary antibodies.

### In Vivo Imaging System

For biodistribution studies, mice were intravenously injected with Cy5.5, Cy5.5‐NPs, or CD38‐Cy5.5‐NPs at a dose of 0.6 mg kg^−1^. Mice were anesthetized and imaged at predetermined time points using an in vivo imaging system (IVIS Lumina XRMS, USA). After 24 h, major organs and bones were isolated and imaged. Fluorescence signals were quantified using Living Image Software (IVIS Lumina XRMS, USA).

### Statistical Analysis

All experiments included a minimum sample size of *n* ≥ 3. Statistical analyses were performed using GraphPad Prism 9. Data were presented as the mean ± standard deviation. Two‐tailed Student's *t*‐test was used for comparisons between two groups. Survival analysis was performed using Kaplan–Meier survival plots and log‐rank tests. A *p*‐value of less than 0.05 was considered statistically significant.

## Conflict of Interest

The authors declare no conflict of interest.

## Author Contributions

R.J. and Y.L. contributed equally to this work. Z.Z. and T.S. designed the research. R.J., Y.L., and J.X. performed the experiments. R.J., X.Z., and Y.X. analyzed the data. R.J., Y.L., and T.S. wrote the manuscript. J.Y. and H.M. provided scientific and technical support. Z.Z., T.S., and C.J. supervised and coordinated all aspects of the research. All authors revised, read, and approved the final manuscript.

## Supporting information



Supporting Information

## Data Availability

The data that support the findings of this study are available from the corresponding author upon reasonable request.
