# Supplementary material for: A ROS‐Responsive Dual‐Targeting Drug Nanocarrier Serving as a GSI Synergist and Ferroptosis Sensitizer for T‐Cell Acute Lymphoblastic Leukemia
Source: Adv Sci (Weinh). 2025 May 31;12(31):e05087. doi: 10.1002/advs.202505087 (PMC12376615; doi:10.1002/advs.202505087)
Supplement: Supplementary file 1 — Supporting Information [file ADVS-12-e05087-s001.docx]

**Supplementary Figures**

**
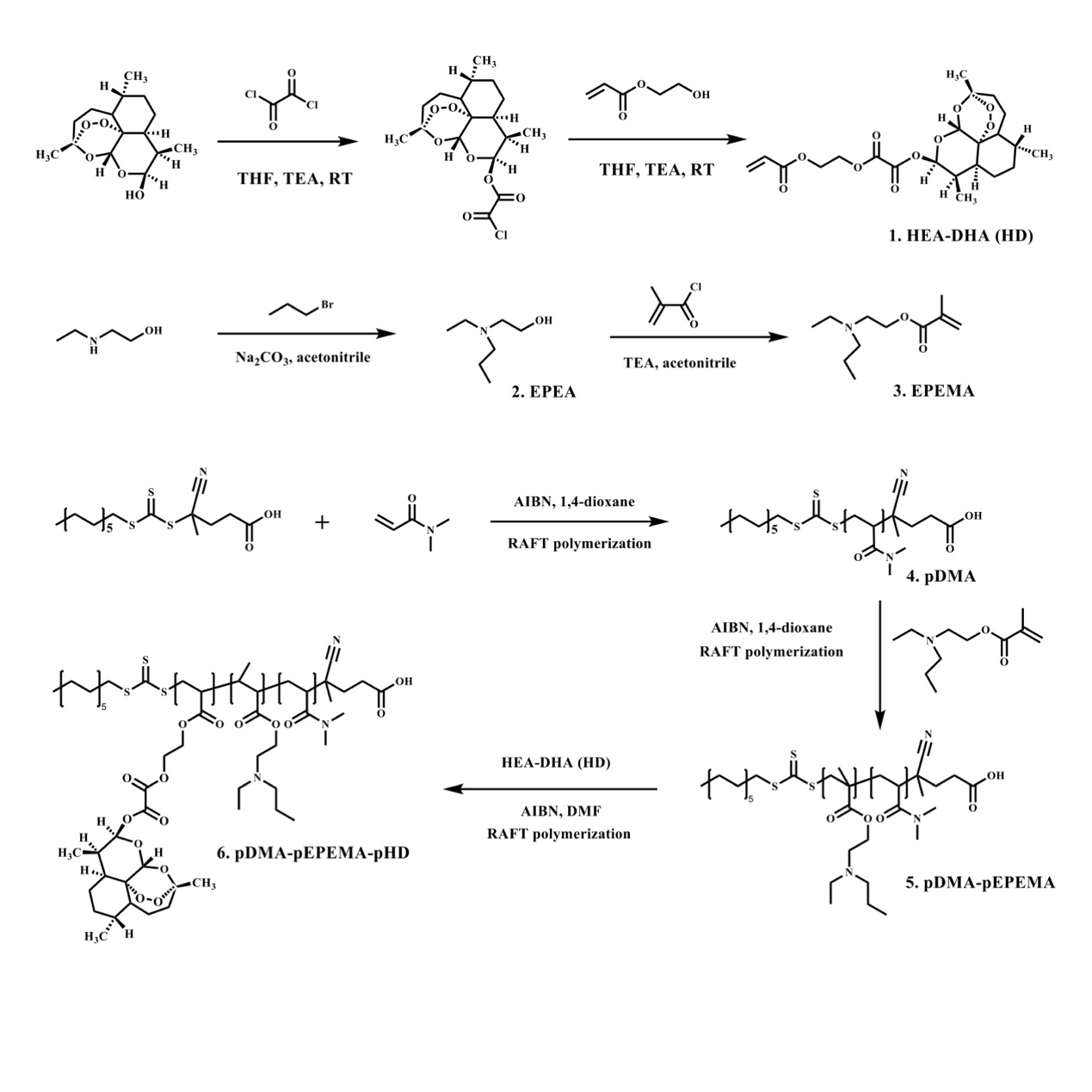
**

**Supplementary Figure 1.** The synthetic methods and routes of ROS responsive polymer pDMA-pEPEMA-pHD. The synthesis of monomers, including the HEA-DHA (HD) and EPEMA. The polymerization of polymers includes the pDMA, pDMA-pEPEMA and pDMA-pEPEMA-pHD.

**
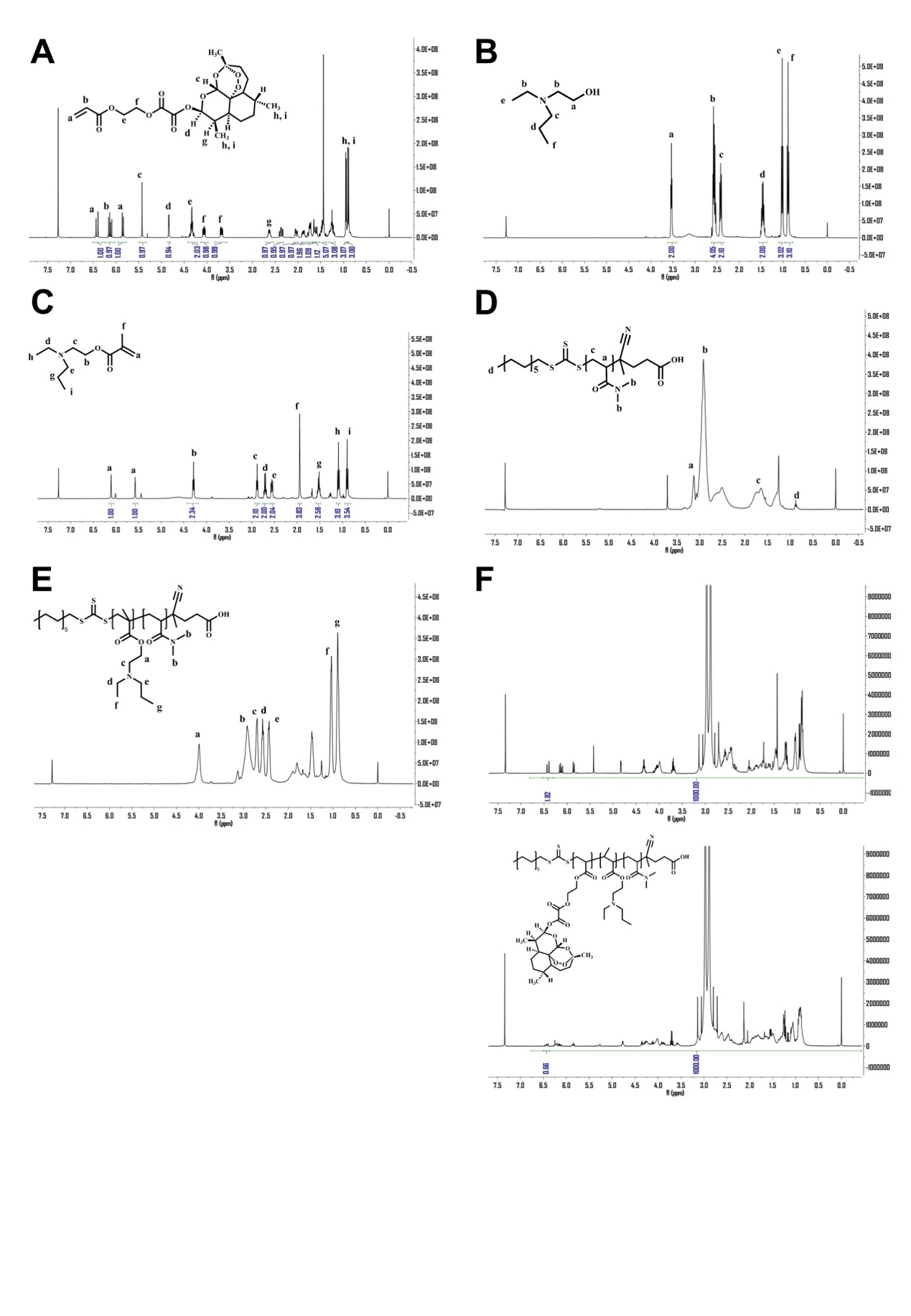
**

**Supplementary Figure 2. The synthesis of ROS responsive polymer pDMA-pEPEMA-pHD.**

(A) ^1^H-NMR spectra of HD. (B) ^1^H-NMR spectra of EPEA. (C) ^1^H-NMR spectra of EPEMA. (D) ^1^H-NMR spectra of pDMA. (E) ^1^H-NMR spectra of pDMA-pEPEMA. (F) ^1^H-NMR spectra of pDMA-pEPEMA-pHD before (T_0_) and after (T_N_) polymerization.

**
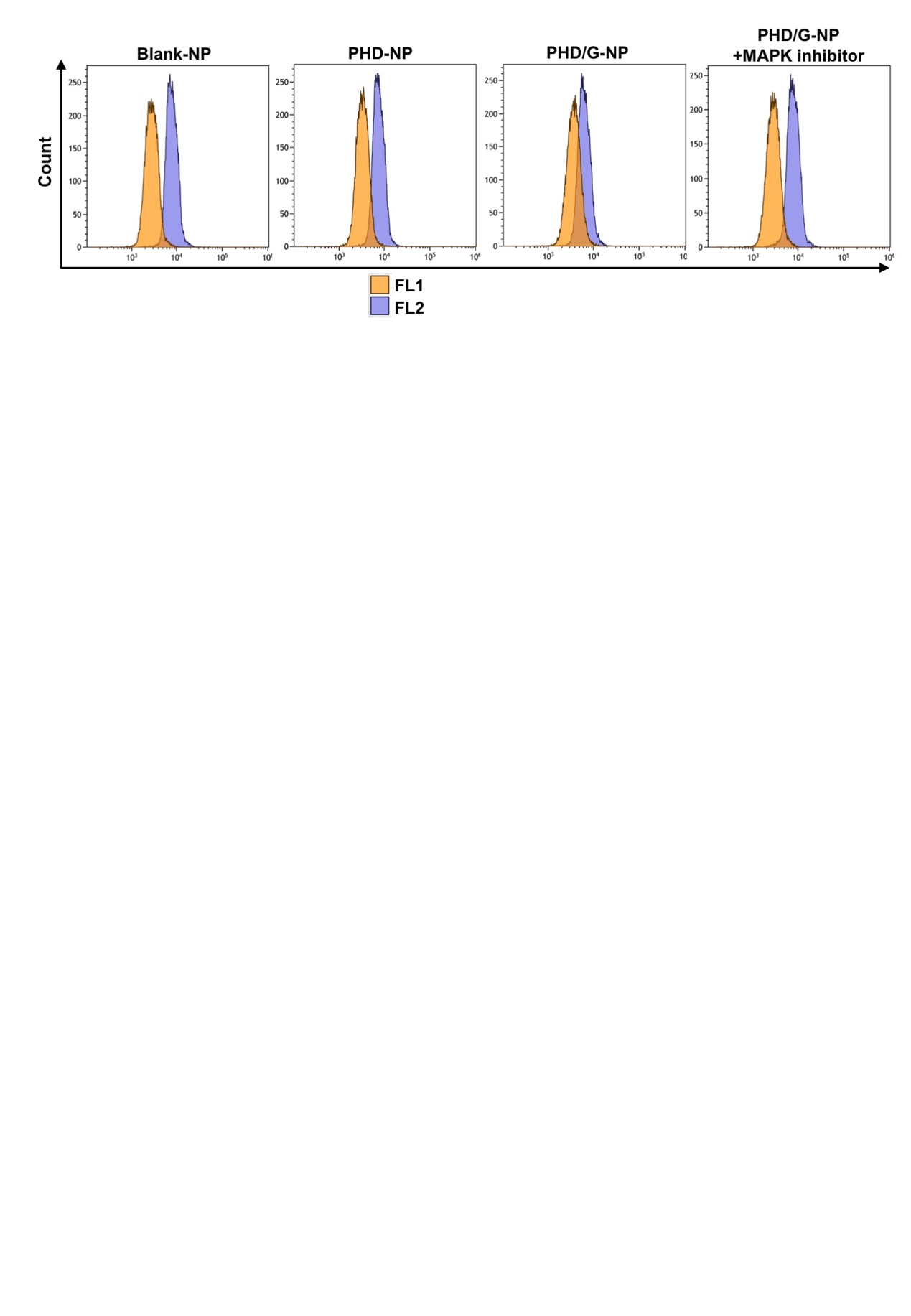
**

**Supplementary Figure 3. The Mechanism PHD/G-NPs inducing facilitated ferroptosis.**

Flow cytometric analysis of lipid ROS in T-ALL cells under various treatment by C11 BODIPY.

**
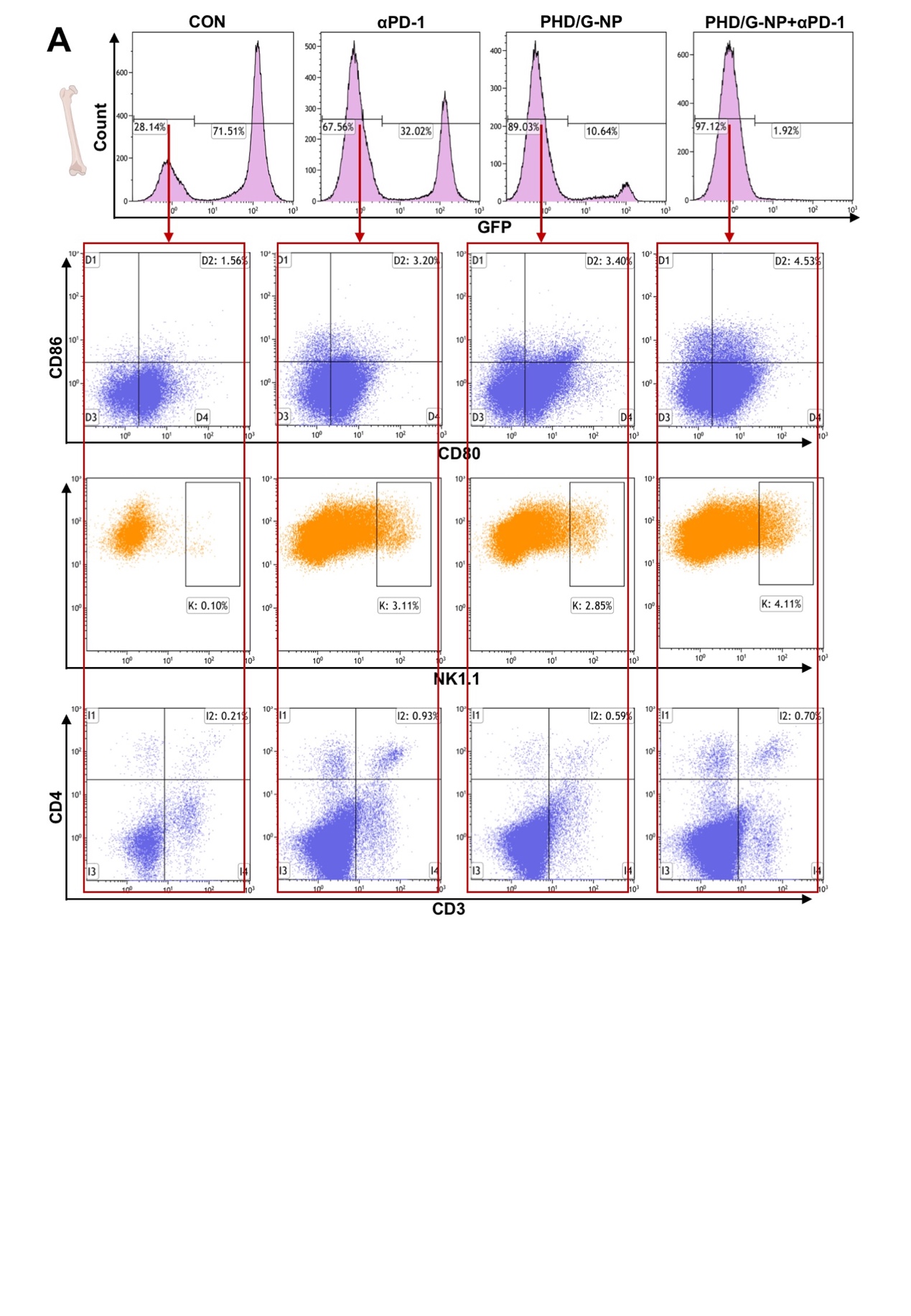
**

**
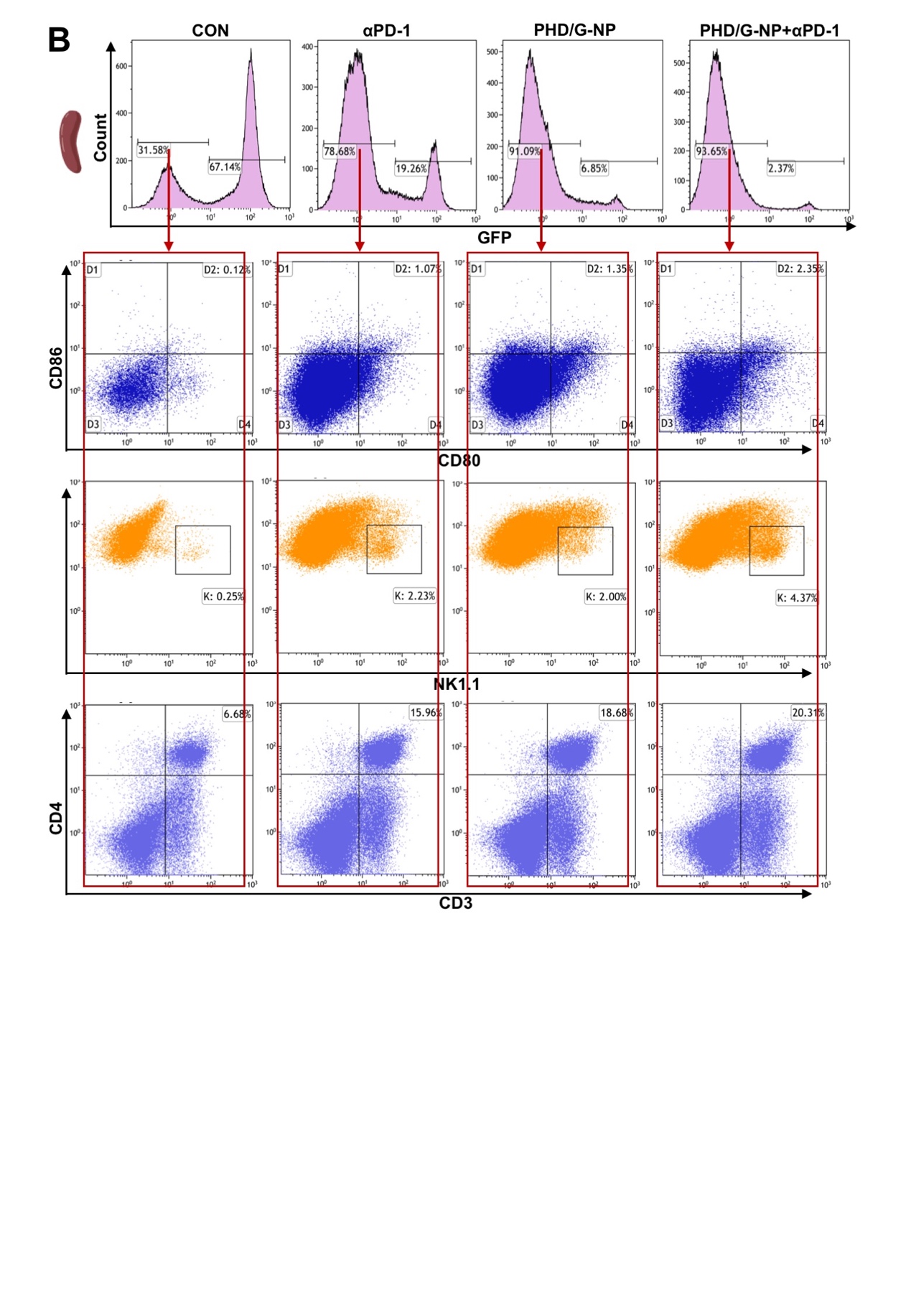
**

**Supplementary Figure 4. PHD/G-NPs synergistically activate anti-tumor immune responses combined with αPD-1 in vivo.**

(A) Representative flow cytometric analysis of the bone marrow DC cells (CD80+CD86+), NK cells (NK1.1+) and CD4+ T cells (CD3+CD4+) in nonT-ALL cells (GFP-). (B) Representative flow cytometric analysis of the DC cells (CD80+CD86+), NK cells (NK1.1+) and CD4+ T cells (CD3+CD4+) in the nonT-ALL cells (GFP-) of spleen.

**
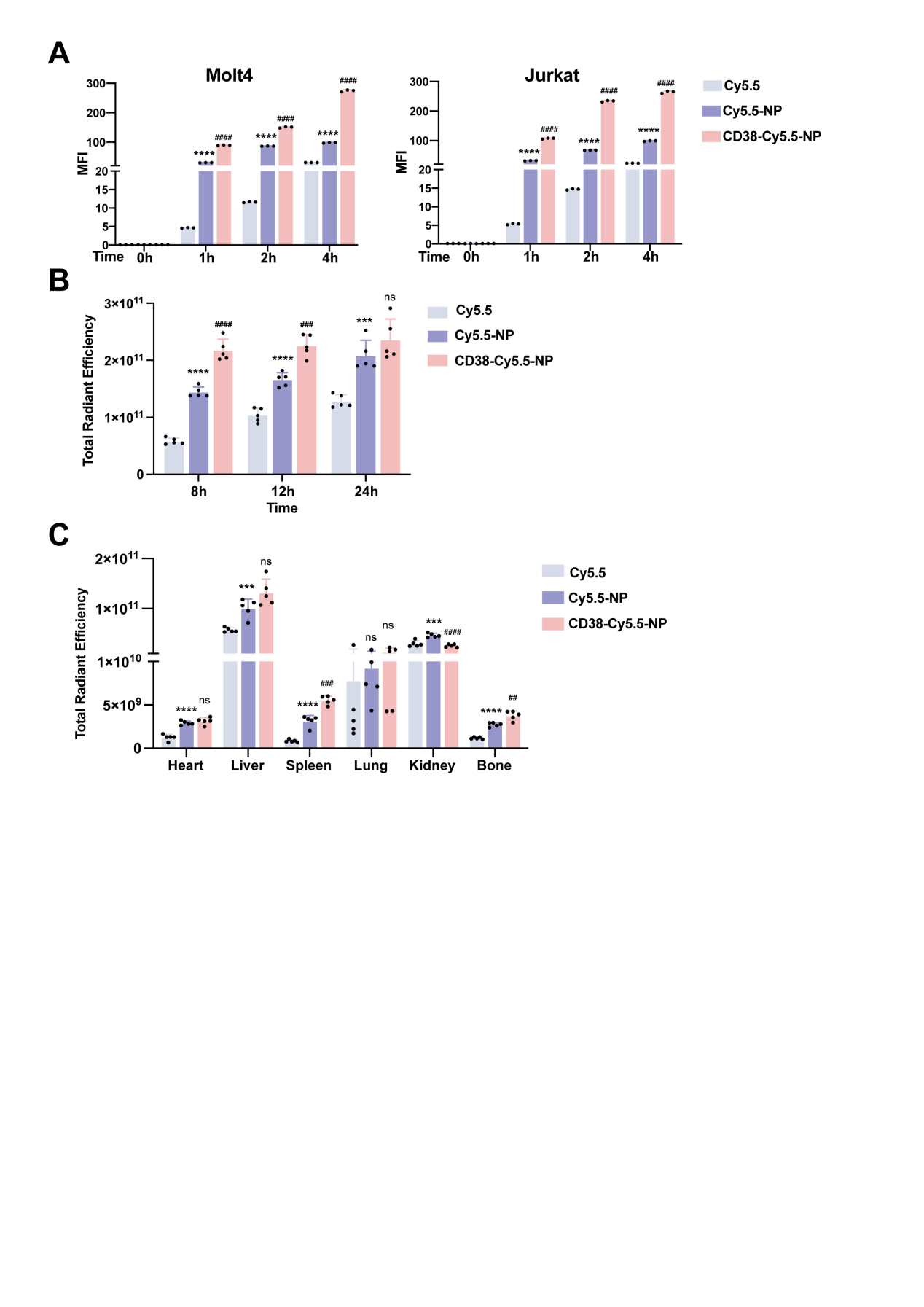
**

**Supplementary Figure 5. The targeted effect of CD38-antibody in T-ALL CDX model.**

(A) Quantitative analysis of mean fluorescence intensity (MFI) in T-ALL cells incubated with Cy5.5 dye, Cy5.5-NPs or CD38-Cy5.5-NPs after indicated time by flow cytometry. (B) Quantitative analysis of Cy5.5 dye intensity in mice treated with Cy5.5, Cy5.5-NPs or CD38-Cy5.5-NPs in Molt4 bearing mice at the indicated time after injection (n = 5). (C) Quantitative analysis of Cy5.5 dye intensity in the indicated organs *ex vivo* at 24 h after injection (n = 5). *, vs. the Cy5.5 group; #, vs. the Cy5.5-NP group. Data represent mean ± s.d. Two-tailed Student’s t-tests were used to assess statistical significance. ns, no significance, *p < 0.05, **p < 0.01, ***p < 0.001, ****p < 0.0001.
